# Transcriptomic profiles of peripheral white blood cells in type II diabetes and racial differences in expression profiles

**DOI:** 10.1186/1471-2164-12-S5-S12

**Published:** 2011-12-23

**Authors:** Jinghe Mao, Junmei Ai, Xinchun Zhou, Ming Shenwu, Manuel Ong, Marketta Blue, Jasmine T Washington, Xiaonan Wang, Youping Deng

**Affiliations:** 1 Department of Biology, Tougaloo College, Tougaloo, MS 39174, USA; 2 School of Computing, University of Southern Mississippi, Hattiesburg, MS 39406, USA; 3 Department of Pathology, University of Mississippi Medical Center, Jackson, MS 39216, USA; 4 Madden Medical Clinic,1071 East Franklin St, Carthage, MS 39051, USA; 5 School of Medicine, University of Mississippi Medical Center, Jackson, MS 39216, USA; 6 Wuhan University of Science and Technology, Wuhan, Hubei 430081, P.R. China; 7 Department of Internal Medicine, Rush University Medical Center, Chicago, IL 60612, USA

## Abstract

**Background:**

Along with obesity, physical inactivity, and family history of metabolic disorders, African American ethnicity is a risk factor for type 2 diabetes (T2D) in the United States. However, little is known about the differences in gene expression and transcriptomic profiles of blood in T2D between African Americans (AA) and Caucasians (CAU), and microarray analysis of peripheral white blood cells (WBCs) from these two ethnic groups will facilitate our understanding of the underlying molecular mechanism in T2D and identify genetic biomarkers responsible for the disparities.

**Results:**

A whole human genome oligomicroarray of peripheral WBCs was performed on 144 samples obtained from 84 patients with T2D (44 AA and 40 CAU) and 60 healthy controls (28 AA and 32 CAU). The results showed that 30 genes had significant difference in expression between patients and controls (a fold change of <-1.4 or >1.4 with a P value <0.05). These known genes were mainly clustered in three functional categories: immune responses, lipid metabolism, and organismal injury/abnormaly. Transcriptomic analysis also showed that 574 genes were differentially expressed in AA diseased versus AA control, compared to 200 genes in CAU subjects. Pathway study revealed that "Communication between innate and adaptive immune cells"/"Primary immunodeficiency signaling" are significantly down-regulated in AA patients and "Interferon signaling"/"Complement System" are significantly down-regulated in CAU patients.

**Conclusions:**

These newly identified genetic markers in WBCs provide valuable information about the pathophysiology of T2D and can be used for diagnosis and pharmaceutical drug design. Our results also found that AA and CAU patients with T2D express genes and pathways differently.

## Background

Type 2 diabetes (T2D) is the most common form diabetes, and it accounts for more than 90% of all diagnosed diabetic cases [1]. It is a heterogeneous and multi-factorial disease. Although the exact pathogenesis has not been fully understood, older age, family history, physical inactivity and race/ethnicity are important risk factors[1,2]. African American (AA) is the ethnicity with the highest incidence and mortality rate of T2D. According to 2010 statistics of adults aged 20 years or older, 15.7 million non-Hispanic whites have diabetes (10.2% of the ethnic group), compared to 4.9 million non-Hispanic blacks (18.7%). After adjusting for population age differences, AAs are 1.8 times as likely to have diabetes as CAUs at similar ages [3-5]. In addition, obesity and immune/inflammatory processes contribute to the onset of T2D [6-9]. For example, elevated levels of circulating IL-1β, IL-6, TNFα, and acute phase proteins in T2D have been extensively described in several studies. That may reflect the activation of innate immune cells by excessive levels of nutrient concentration, including glucose and free fatty acids. In turn, both IL-6 and IL-1β act on the liver to produce a characteristic dyslipidemia in metabolic syndrome, one which manifests as increased very low density lipoprotein (VLDL) and decreased high density lipoprotein (HDL) [10,11]. IL-6 and tumor necrosis factor TNFα also participate in the impairment of insulin signaling in adipocytes [12].

Many clinical and epidemiological studies have been done to elucidate environmental contribution to T2D [13-15]. Microarray analysis will allow us to study genetic contribution and patterns of altered gene expression related to T2D. Thus far, human skeletal muscles, liver, adipose tissues, and pancreatic β-cells [16-22] have been chosen for gene expression assay in T2D. These studies have yielded important insights into the pathogenesis of T2D and its relationship to obesity. For example, adipose tissue gene expression has been found to show more differences between T2D and healthy control than muscle tissue gene expression. Many genes are inflammatory and metabolically important [23]. Because it is difficult to obtain permission from a clinic to use tissue samples such as liver, adipose, and muscle for genetic studies, the peripheral blood could be a convenient source of cells for this study. To date, only one microarray study of human peripheral blood has been reported by Grayson et al [24]. Their finding indicated that genes differentially expressed in T2D have key roles in T-cell activation and signaling, but this study used a limited sample size (n = 6) and lacked ethnicity information. In this study, we measured the up/down regulation of some T2D-related genes using white blood cells from T2D patients, and we compared the gene expression profiles between two ethnic groups. The information obtained from our study would provide further understanding of the pathogenesis of T2D and the disparities in expression profiles between AA and CAU.

## Results

### Characteristics of studied subjects

This study comprised a balanced distribution of the studied subjects in gender and ethnicity: among 60 controls, 28 were AA including 14 females and 14 males, and 32 were CAU including 14 females and 20 males. Among 84 patients with T2D, 44 were AA including 22 females and 22 males, and 40 were CAU including 23 females and 17 males. As compared to AA, CAU had a significantly higher level of blood triglycerides (TG) in both the controls (106 ± 54.3 mg/dl in AA vs. 153 ± 77.8 mg/dl in CAU, p = 0.0009), and the patients (157 ± 128 mg/dl in AA vs. 207 ± 98.3 mg/dl in CAU, p = 0.037). There were no significant differences in other studied clinical parameters between two races (data for ethnicity differences were not shown). As compared to all controls (mixed), the patients group was 4.5 years older, had significantly higher body mass index (BMI), blood TG, and fasting glucose, and had lower high density apolipoprotein (HDL). There were no differences in low density apolipoproteins (LDL) and total cholesterols (Table 1) between controls and T2D patients.

**Table 1:** The clinical characteristics of the study subjects

	Normal controls	Diabetic
	(n = 60)	(n = 84)
Age, yr (mean ± SD)	58.5 ± 16.1	63 ± 13
Sex (female/male)	28/32	45/39
Race	32 Caucasian	40 Caucasian
	28 African American	44 African American
Body mass index (kg/m^2^)	30.1 ± 7.3	34.2 ± 8.4**
Triacylglycerol (mg/dL)	134 ± 76.9	186 ± 113.8**
HDL cholesterol (mg/dL)	56.6 ± 17.5	49.1 ± 15.4**
LDL cholesterol (mg/dL)	112 ± 44.8	109.8 ± 36.5
Total cholesterol (mg/dL)	197 ± 44.8	195 ± 46.8
Glucose (mg/dL)	88.8 ± 10.8	142.7 ± 56.8***

Data are mean ± SD. Statistical analyses were performed using the Student's t-Test. **p < 0.05 ***p < 0.001, values are based on differences from controls.

### Identification of differentially expressed genes between all T2D patients and healthy controls

Whole human genome (4 × 44K) oligo-microarrays of peripheral WBCs from 60 healthy subjects and 84 T2D subjects were performed. Of 41,000 probes available on the Agilent arrays, we identified 30 genes that were differentially expressed (a fold change of <-1.4 or >1.4 with a P value <0.05). Six known genes were up-regulated, including erythroid associated factor (*ERAF*), delta-aminolevulinate synthase 2 (*ALAS2*), oxysterol binding protein 2 (*OSBP2*), carbonic anhydrase I (*CA1*), serine/threonine/tyrosine kinase 1 (*STYK1*), and Zinc finger protein 2 (*ZIC2*). Sixteen known genes were down-regulated, including G0/G1 switch 2 (*GOS2*); telomerase-associated protein 1 (*TEP1*); prostaglandin-endoperoxide synthase 2 (*PTGS2*); interleukin 4 (*IL4*); interleukin 8 (*IL8*); interferon alpha-inducible protein 27 (*IFI27); *interferon-induced protein with tetratricopeptide repeats 3 (*IFIT3*); interferon-induced protein with tetratricopeptide repeats 2 (*IFIT2*); tumor necrosis factor alpha-induced protein 6T (*NFAIP6*); radical S-adenosyl methionine domain containing 2 (*RSAD2*); apolipoprotein B mRNA editing enzyme, catalytic polypeptide-like 3A (*APOBEC3A*); ATP/GTP binding protein-like 4 (*ABGL4*); ATP-binding cassette sub-family A transporter (*ABCA1*); epithelial stromal interaction 1 isoform 2 (*EPSTI1*); Ephrin type-A receptor 6 (EPHA6); and leucine rich repeat neuronal protein 3 (*LRRN3*). Most of these genes belonged to three functional categories: immune and inflammatory responses, such as *IL-4, IL-8, TNFAIP6*, and *PTGS2*; lipid and glucose metabolism, such as *ABCA1, OSBP2*, and *GOS2*; and organismal injury and abnormalities, such as *APOBEC3A*. Thus, these genes might be the genetic signatures for T2D.

### Ethnic differences in gene expression profiles

**Figure 1:**
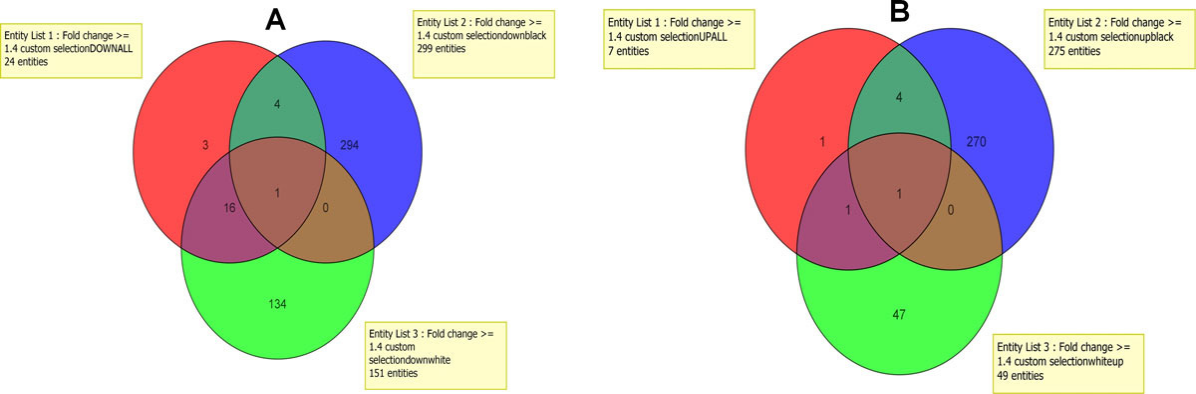
**Venn diagrams for down-regulated and up-regulated genes in all mixed, AA and CAU T2D patients**. a) Significantly down-regulated genes in mixed T2D patients are represented in red, AA in blue, and CAU in green. (b) Significantly up-regulated genes in mixed T2D patients are represented in red, AA in blue, and CAU in green.

In order to study ethnic disparities, gene expression profiles between AA and CAU participants were analyzed and compared. Based on the same criteria described before, many more genes were found to be differentially expressed. 574 genes were differentially expressed in AA (28 healthy controls and 44 T2D patients) compared to 200 genes in CAU (32 healthy controls and 40 T2D patients). In the AA group, there were 294 down-regulated and 269 up-regulated genes in T2D patients; however, we only saw 134 down-regulated and 47 up-regulated genes in CAU T2D patients (Figure 1a and 1b). It was obvious that AA T2D patients had 2-6 times more altered genes than CAU T2D patents. Table 2 summarizes the top 10 up-regulated and down-regulated genes in different ethnic groups (a fold change of <-1.4 or >1.4 with a P value <0.05). Overlap analysis showed that AA had an entirely different spectrum of genetic signatures for T2D, because only one down-regulated gene and one up-regulated gene were common between the two ethnicities (Figure 1a and 1b).

**Table 2:** Differentially expressed top 10 genes in two ethnic groups

African American				Caucasian			
	
Up-regulated		Down-regulated		Up-regulated		Up-regulated	
			
Gene name	Fold*	Gene name	Fold*	Gene name	Fold*	Gene name	Fold*
IRX5	1.9	HLA-DQA1	-2.4	OLFM4	1.9	DDX3Y	-3.1
THC2540257	1.8	MED18	-2.3	ENST00000382691	1.8	IFI27	-2.5
THC2521898	1.8	THC2667190	-1.8	CEACAM8	1.6	DB160230	-2.4
WDR49	1.8	GNL3L	-1.8	WIF1	1.6	CYorf15B	-2.4
RNF150	1.8	CENPI	-1.8	AI796347	1.6	BTNL8	-2.3
AK095945	1.8	RNF125	-1.7	HLA-DQA2	1.6	EGR1	-2.3
AA579646	1.8	BU627449	-1.7	W60781	1.5	BQ213856	-2.1
THC2576067	1.7	ITIH5	-1.7	OR51B2	1.5	EGR2	-2.0
BQ934520	1.7	AK097130	-1.7	A_32_P58705	1.5	SIGLEC1	-1.9
A_24_P533990	1.7	ATM	-1.7	CPSF2	1.5	IFIT1	-1.8

* Fold was the ratio of T2D patient to control in each group. The differences in all listed genes.

between T2D patients and controls were <0.05.

### Ethnic differences in pathway enrichment analysis

**Figure 2:**
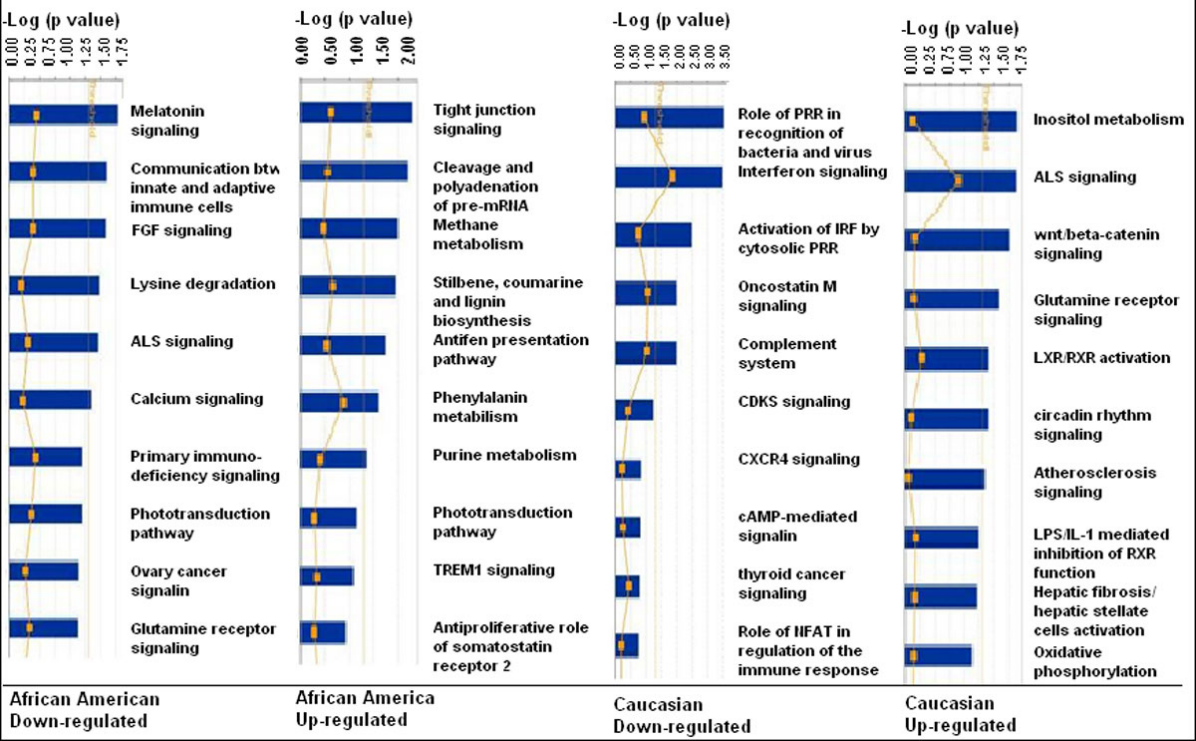
**Pathway analyses using specifically regulated genes in AA and CAU**. The top ten pathways were selected to present for specifically up- or down-regulated genes in AA and CAU patients. The significantly different genes were chosen to run the Ingenuity pathway tool. The bigger the -log(p-value) of a pathway is, the more significantly the pathway is regulated. The threshold lines represent a p value with 0.05.

Gene expression profiling study showed ethnic disparities in this study cohort as described above. Because one pathway usually involves many different genes, it is not clear whether the differentially expressed genes in the two ethnic groups involved the same pathways. Using the tool of Ingenuity Canonical Pathways Analysis, the differentially expressed genes in two ethnic groups were clustered into various pathways. The top 10 down-regulated and up-regulated pathways were identified for T2D patients in AA and in CAU as shown in Figure 2. Similar to the results of differentially expressed single genes, the two T2D patient groups had different spectra of down- and up-regulated pathways. For example, significant enrichments of down-regulated pathways in AA patients were "communication between innate and adaptive immune cells" and "primary immunodeficiency signaling", whereas those in CAU patients were "interferon signaling" and "complement system" pathways. The main up-regulated pathways in AA included "inositol metabolism", "Wnt/β-catenin signaling", and "LXR/RXR activation", but for CAU they were "tight junction signaling", "cleavage and polyadenyation of pre-mRNA", and "methane metabolism".

### Microarray validation by real-time PCR

**Figure 3:**
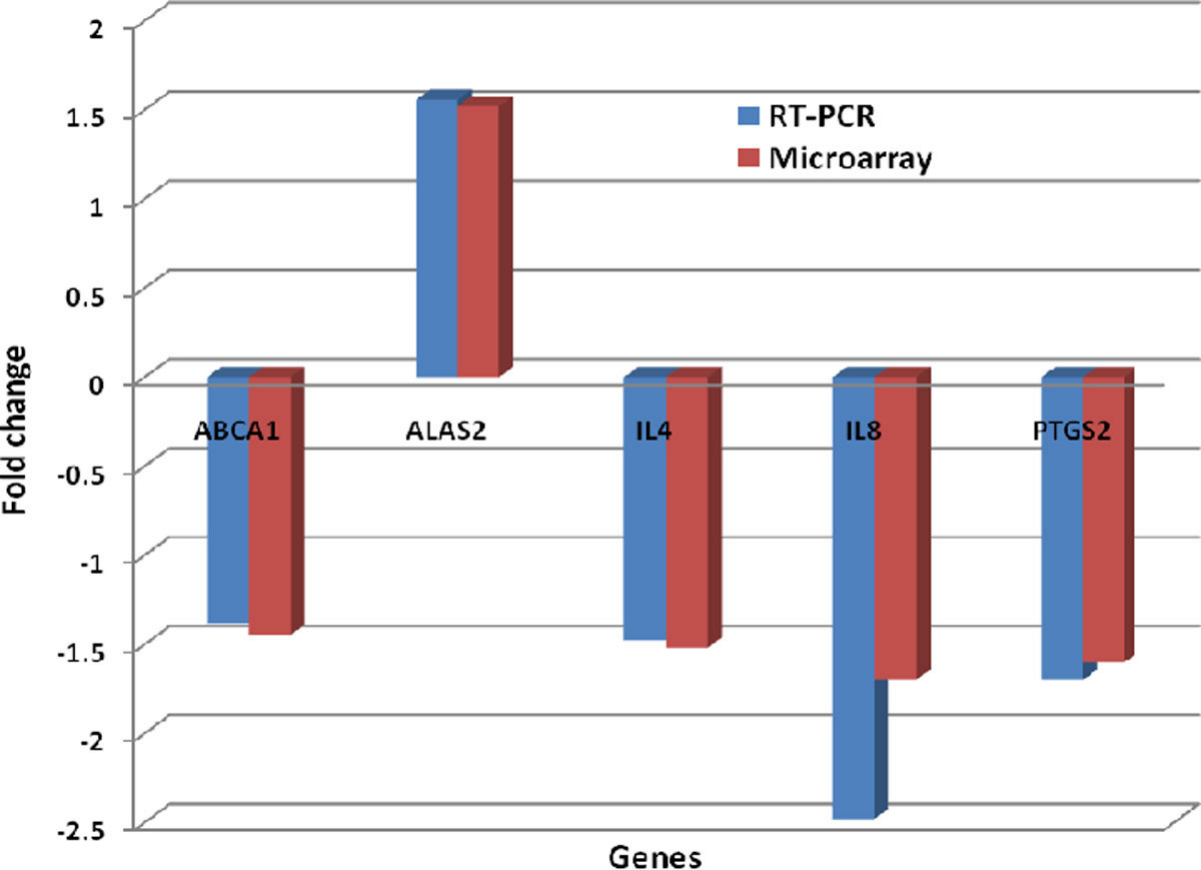
**Expression concordance between microarray and quantitative real-time PCR assays**. The y-axis represents the ratio of expression values between the T2D and control groups.

To confirm the accuracy of the results in microarray study, real-time PCR were performed on 5 randomly selected genes (*ABCA1, IL-4, IL-8 and PTGS2 *except *ALAS2) *that were found to be differentially expressed in microarray analysis. All of these genes have a known function in glucose and lipid metabolism and in immune responses. The fold changes for the transcripts of these genes were normalized to the transcripts of glyceraldehyde-3-phosphate dehydrogenase (*GAPDH*); information of primers for the 5 selected genes and *GAPDH *was shown in table 3. The fold changes of those five gene expressions, as determined by microarray and real-time PCR, are shown in Figure 3. The results validated our microarray data.

## Discussion

Previous studies of gene expression in T2D have been restricted to adipose tissue and muscle or to animal models with limited sample size [16-22]. Before our study, the comparison of blood gene expression in AA and CAU with T2D patients was lacking. Our analysis of gene profiling in peripheral WBCs from a larger number of sample and ethnic groups begins to fill the gaps. We showed that 30 genes are differentially expressed in T2D patients when compared with healthy controls. Furthermore, we demonstrated that different gene expression signatures are present in AA and CAU patients.

In T2D, we see increased expression of six genes in which five of those have not been previously reported in gene expression studies. *ERAF *is associated with haemoglobin stabilization [25], *ALAS2 *is involved in immune responses [26], *OSBP2 *in lipid metabolism [27], *STYK1 *in signal transduction [28], and *ZIC2 *in transcriptional regulation [29]. Carbonic anhydrase I, encoded by *CA1*, has been reported to be progressively increased in the urine of T2D patients [30]. Interestingly, in our study, we found no evidence for up-regulation of classical inflammatory markers including the interleukin family, the TNF family, or interferon family which have been shown in the literature [5-9]; however, a panel of genes belonging to those families is down-regulated in our study cohort, including *PTGS2, IL4, IL8, IFI27, IFIT3, IFIT2, TNFAIP6, RSAD2*, and *AGBL4*. All of those are associated with the interferon signaling pathway, immune signaling pathways involved in inflammatory processes [31]. Our results suggested that the innate immunity was actually compromised, and significantly lower inflammation was observed in T2D patients. These novel findings are inconsistent with the results from other studies, which may indicate an important difference in the pathophysiology of T2D between the tissues. A recent study [32] also showed that elevated levels of circulating inflammatory markers in T2D may not necessarily reflect the degree of inflammation in individual tissues because of the mass difference. In summary, peripheral WBC gene expression profiling may not mirror the changes of gene expression within a specific tissue such as adipose or muscle.

In addition to inflammatory markers, we found down-regulation of several genes (for example *ABCA1 *and *GOS2) *which are reported to be associated with lipid metabolism and T2D in gene knockout cell lines and/or animal models [33-35]. For example, recent studies have shown that ATP-binding cassette transporters ABCA1 and ABCG1 play important roles in macrophage cholesterol efflux to serum or HDL in macrophage foam cells [33]. Mice lacking ABCA1 and ABCG1 accumulate inflammatory macrophage foam cells in various tissues such as lung, liver, spleen, or myocardium [34]. Therefore, ABCA1 may have a unique role in dampening inflammation. Consistent with their finding, our data showed that ABCA1 gene was down-regulated in T2D patients. In addition, we also see that patients had significant lower HDL when compared with control subjects (Table 1). To our knowledge, this is the first evidence that lower expression of ABCA1 decreases the cholesterol efflux from macrophage, resulting in less HDL formation in T2D patients. In turn, the anti-inflammatory and immunosuppressive functions of ABCA1 are diminished.

Yang *et al.*, [35] recently reported that G0S2 expression was significantly reduced in white adipose tissues (WAT) of *db/db *mice and high-fat-fed wild-type mice. They speculated that decreased insulin sensitivity of adipocytes may lead to the down-regulation of G0S2, but they are not sure whether expression of G0S2 is also decreased in obese humans. Our gene expression profiling provided strong evidence that GOS2 was down-regulated in T2D patients, who also had significant higher BMI compared tohealthy controls.

Type 2 diabetes (T2D) is a complex disease. Although the exact pathogenesis has not been fully understood, family history, physical inactivity, socioeconomic condition, and race/ethnicity contribute to the onset of T2D [13-15]. AAs have the highest incidence and mortality rate of T2D [5]. To better understand the health disparity, we compared the clinical parameters and expression profiles of peripheral WBCs from AA and CAU. As described previously, CAU had a significantly higher level of blood triglycerides (TG) in both the controls and the patients. There was no significant difference in other clinical parameters between two races. However, as shown in Figure 1, AA T2D patients had 2-6 times more altered genes than CAU T2D patients. In AAs, there were 294 down-regulated and 269 up-regulated genes in T2D patients; however, we only saw 134 down-regulated and 47 up-regulated genes in CAUs. The differences were also observed in the top 10 up-regulated and down-regulated genes from the two ethnic groups shown in table 3. None of the up- or down-regulated genes overlapped between AAs and CAUs. In agreement with gene expression differences, pathway enrichment study further suggested that AAs have different pathophysiological mechanisms of T2D from CAU. For example, significant enrichments of top 10 down-regulated pathways in AA patients included "communication between innate and adaptive immune cells" and "primary immunodeficiency signaling", whereas that in CAU patients were "interferon signaling" and "complement system" pathways. The main up-regulated pathways in AA were "inositol metabolism", "Wnt/β-catenin signaling" and "LXR/RXR activation", but these pathways were not significantly altered in CAU. Taken together, differences between AA and CAU participants in expression profiles parallel differences between these groups in pathway enrichment data. Ethnic differences in TG cannot explain ethnic disparities. The possibility that CAU persons have systematically higher levels of TG warrants further study.

**Table 3:** Primers for quantitative real time PCR experiments

Gene	Forward primer	Reverse primer
*ABCA1*	5'-TGTCCAGTCCAGTAATGGTTCTGT-3'	5'-CGAGATATGGTCCGGATTGC-3'
*ALAS2*	5'-GCCGCCGAATTCAAACTTGAATTTTCAT-3'	5'GCCGCCGAATTCGCCCTTCTGTACTGTTT-3'
*IL4*	5'-CCGTAACAGACATCTTTGCTGCC-3'	5'-GAGTGTCCTTCTCATGGTGGCT-3'
IL8	5'-GAGAGTGATTGAGAGTGGACCAC-3'	5'-CACAACCCTCTGCACCCAGTTT-3'
*PTGS2*	5'-TATGTTCTCCTGCCTACTGGAA-3'	5'-GCCCTTCACGTTATTGCAGATG-3'

## Conclusions

In summary, through the analyses of clinical parameters, expression profiles of T2D, and ethnic differences of gene expression and pathway enrichment, we found that the innate immunity was compromised and significantly lower inflammation was associated with T2D. The newly identified *ABCA1 *and *GOS2 *in human could be used as genetic markers for diagnosis and pharmaceutical drug design. Our results also indicated that genes and pathways are differentially expressed between AA and CAU T2D patients.

## Materials and methods

### Subjects and clinical laboratory data

The study was approved by the Institutional Review Board of Tougaloo College. All subjects provided written informed consent for this study. T2D was diagnosed based on American Diabetes Association (ADA) guidelines [5] and characteristic symptoms of diabetes: a higher BMI and a fasting plasma glucose > 126 mg dl^-1 ^or a 2 h plasma glucose during an oral glucose tolerance test of > 200 mg dl^-1^. A total of 144 blood samples from healthy controls (n = 60, 32 Caucasians and 28 African Americans), and T2D (n = 84, 40 Caucasians and 44 African Americans) were collected. All subjects were evaluated by age, sex, ethnicity, body mass index (BMI), triacylglycerol (TG), high-density lipoprotein (HDL), low-density lipoprotein (LDL), total cholesterol (TC), and glucose levels.

### RNA isolation

Total RNA from 8-10 mLs peripheral blood WBCs was obtained using LeukoLock™ Total RNA system (Ambion Inc, Austin, TX) according to the manufacturer's instructions. The quantity and quality of the isolated RNA were determined by Nanodrop spectrophotometry and Agilent 2100 Bioanalyzer (Agilent Technologies, Santa Clara, CA).

### Microarray experiments

Gene expression profiling was conducted using Agilent Whole Human Genome1 (4 × 44K) Oligo arrays with ~20,000 genes represented (Agilent Technologies, Palo Alto, CA). Each sample was hybridized against a human universal RNA control (Stratagene, La Jolla, CA). 500 ng of total RNA was amplified and labeled using the Agilent Low RNA Input Fluorescent Linear Amplification Kit, according to manufacturer's protocol. For each two color array, 850 ng of each *Cy5*- (universal control) and *Cy3*-labeled (sample) cRNA were mixed and fragmented using the Agilent *In Situ *Hybridization Kit protocol. Hybridizations were performed for 17 hours in a rotating hybridization oven according to the Agilent 60-mer oligo microarray processing protocol prior to washing and scanning with an Agilent Scanner (G2565AA, Agilent Technologies, Wilmington, DE). Arrays were processed and background corrected with default settings for all parameters with the Agilent Feature Extraction software (v.9.5.3.1).

### Microarray data analysis

Microarray data analyses were processed with GeneSpring version 7.0 and 10.0. The sample quality control was based on the Pearson correlation of a sample with other samples in the whole experiment. If the average Pearson correlation with other samples was less than 80%, the sample was excluded for further analysis. If the scanned intensity was less than 5.0 for a probe, it was transformed to 5. A perchip (within) array normalization was performed using 50 percentile values of all the probe values in the array. Per gene (between) array normalization was also applied using the median value of a gene across all samples in the experiment. Probe features were first filtered using flags. A "present" or "absent" flag was defined using the Agilent *Feature Extraction 9.5.1 *software. Only a probe that had present flags in at least 50% samples of all the arrays was kept for further analysis. Data were subsequently log (base 2) transformed for statistical analysis. To identify differentiated genes between two groups (e g. normal *vs*. diabetes), an un-paired t-test with cut off p value 0.05 was applied to compare control samples and TNT exposed samples. In addition, 1.4 fold changes were applied to identify more significantly regulated genes.

### Gene functional analysis and pathway analysis

Significantly regulated probes were employed for one way hierarchical clustering (only cluster genes) or two-way hierarchical clustering (clustering both genes and samples) using GeneSpring 7.0 and/or 10 (Agilent Technologies, Foster City, CA, USA). A Pearson correlation with average linkage was applied for the clustering. Gene functional categories were classified according to Gene Ontology (GO) [36] using The Database for Annotation, Visualization and Integrated Discovery (DAVID) [37,38] and Gofetch [39] tools. A Gene Ontology functional term enrichment p value less than 0.05 was considered significant. Pathway analysis was performed using the Ingenuity canonical pathways analysis tool. Similar to GO analysis, a pathway with an enrichment p value less than 0.05 was considered to be a significantly regulated pathway (Ingenuity Systems, Inc., Redwood City, CA). Gene networks were constructed based on the Ingenuity knowledge base. A score was assigned to a network according to the fit of the original set of significant genes. This score reflects the negative logarithm of the p value that indicates the likelihood of the focus genes in a network being found together due to random chance [40].

### Real-time PCR

To validate the microarray data, a real-time PCR assay was performed using Brilliant II SYBR^® ^Green system for five genes. Samples were selected from the original microarray experiments for further RT-PCR testing based on sufficient RNA remaining. Glyceraldehyde-3-phosphate dehydrogenase (GAPDH) expression was used as an internal control and the relative expression of each gene was determined by the ΔΔCt method, comparing expression of test gene to an average GAPDH, and then comparing the T2D group versus healthy control group. The primers used for cDNA amplification are shown in Table 3.

## Competing interests

The authors declare that they have no competing interests.

## Authors' contributions

JM conceived of the study, and contributed to its design and coordination and drafted the manuscript; JA analyzed microarray data and contributed to the writing of the manuscript. XZ made substantial contributions to conception and design, and has been involved in revising it critically for important intellectual content. MB and MS processed the samples, isolated total RNA, and conducted the array hybridizations and part of the data organization. MO provided clinical samples and other laboratory data. JTW participated in clinical data analysis and English proofreading. YD initiated the project, analyzed microarray data and revised the manuscript. All authors approved the final manuscript. XW participated in clinical discussion and helped to improve the manuscript.
